# Blended learning course for ultrasound-guided diagnostic skills: a design-based research study

**DOI:** 10.3389/fmed.2025.1680563

**Published:** 2026-01-09

**Authors:** Rashmi Ramachandran, Nishkarsh Gupta, Kritika Sharma, Mohit Kumar Joshi, Suhani, Karan Madan, Saurabh Mittal, Satyavir Yadav, Aseem B, Niraj Kumar, Sanjeev Kumar, Niraj Nirmal Pandey, Anju Gupta, Ambuj Roy

**Affiliations:** 1 Department of Anaesthesiology, Pain Medicine and Critical Care, All India Institute of Medical Sciences, New Delhi, India; 2 Department of Onco-Anaesthesia and Palliative Medicine, Dr BRA IRCH, All India Institute of Medical Sciences, New Delhi, India; 3 Department of Surgical Disciplines, All India Institute of Medical Sciences, New Delhi, India; 4 Department of Pulmonary, Critical Care and Sleep Medicine, All India Institute of Medical Sciences, New Delhi, India; 5 Department of Cardiology, All India Institute of Medical Sciences, New Delhi, India; 6 Department of Neuroanaesthesiology and Critical Care, All India Institute of Medical Sciences, New Delhi, India; 7 Department of Cardiovascular Radiology and Endovascular Interventions, All India Institute of Medical Sciences, New Delhi, India

**Keywords:** design-based research, ultrasound education, instructional design, blended learning, medical education research

## Abstract

**Background:**

Simulation-based training has emerged as a critical modality in medical education, particularly for areas like point-of-care ultrasound. The effectiveness of the training is heavily influenced by instructional design. Design-based research offers a framework to iteratively develop, implement, and refine educational interventions, particularly in real-world settings, ensuring both practical and theoretical foundation.

**Objective:**

This study employed a design-based research approach to develop, evaluate, and implement a blended ultrasound education program for in-service medical professionals, integrating asynchronous e-learning with high-fidelity simulation.

**Methods:**

The study was conducted at a single academic institution and involved 51 practicing healthcare professionals. The course was designed, including needs assessment, iterative curriculum development, and multiple cycles of implementation and evaluation. Online module performance, pre- and post-test assessments, and subgroup analysis of curriculum completers were used to evaluate learning outcomes. Instructional decisions were revised based on observed performance trends. Suitable interventions in the design framework were also planned according to all the feedback and data captured.

**Results:**

Participants showed a statistically significant improvement in knowledge post-intervention (mean score increase: 10.24%, *p* = 0.0033). Lung and ocular ultrasound modules showed low variability and high scores, according to module-wise analysis, suggesting good instructional design. Subgroup analysis confirmed the significance of structured progression by showing a correlation between full curriculum engagement and improved performance and consistency. The cardiac ultrasound module showed high variability and lower mean scores, guiding a redesign recommendation for scaffolded instruction.

**Conclusion:**

The design-based approach enabled the systematic development of the ultrasound-guided diagnostics course, ensuring alignment between instructional strategies and learner needs. This study highlights the value of evidence-informed design in enhancing clinical competency in ultrasound procedures and offers a scalable simulation-integrated model for future medical education.

## Introduction

Worldwide, the use of digital technology for learning and education has grown in just over a decade in healthcare (1). The method “see one, do one, teach one” was once a cornerstone of hands-on clinical education, but it is now increasingly viewed as outdated and insufficient in the context of modern healthcare needs (2). Advances in medical knowledge, patient safety standards, and educational tools have led to a shift toward more structured and supervised learning in dealing with these challenges (3). Medical education occupies a pivotal position in these developments, as it is tasked with implementing optimal instructional methodologies to guide the transformation of novice learners into proficient healthcare practitioners (4). Within this framework, simulation-based training has become a core component in equipping novice learners with the competencies required for clinical practice in a risk-free environment (5).

Ultrasound is emerging as an indispensable modality in clinical practice, particularly in point-of-care settings, due to its favorable safety profile, portability, and real-time imaging capabilities, which contribute to enhanced diagnostic accuracy and improved clinical decision-making along with X-ray, CT, and MRI, decisive for differential diagnostics (6). In recent years, there has been a concerted effort to incorporate ultrasound training into medical school curricula and the clinical education of resident physicians. Accordingly, mastery of this technique is essential for delivering efficient, high-quality, and patient-specific care. Use of simulators to learn US-guided diagnostic skills is a novel innovation. Simulation facilitates prompt feedback, allowing healthcare providers to improve task execution by incorporating lessons learned from mistakes or challenges faced during earlier practice attempts (7).

Simulation is being rapidly adopted into professional assessment and certification maintenance processes worldwide. The integration may further enhance these initiatives by enabling the evaluation of performance at scale, aligning with the iterative, data-informed nature of design-based research (7). Research on technology-enhanced simulation training has highlighted that specific instructional design features can impact the learning outcomes, with distinct components playing a crucial role in shaping the effectiveness of training (8). An example of such a technology enhancement can be the use of blended learning for simulation-based US training. To get insights into such a format of training module, the best method would be to conduct a “design-based research.” A foundational feature of the design-based research cycle is that it should produce measurable change in the understanding of the students, enabling participants to review, clarify, and consolidate the knowledge and insights gained, all the while modifying the training activity based on the iterations gained from the conduct of the activity itself (9).

The concept of blended learning has become a firmly established teaching methodology in medical education, particularly as digital transformation reshapes instructional strategies (10). This study aimed to evaluate the effectiveness of integrating a pre-course e-learning module combined with a post-course hands-on workshop. The course was facilitated by an expert faculty panel. We employed a design-based research approach to develop, implement, and refine an integrated simulation-based ultrasound training program. Multiple levels of feedback, both from learners and faculty, were incorporated iteratively to consolidate the program and improve learning. The objective is to describe how specific instructional design features influence learning outcomes, clinical competency, and learner confidence, with a focus on optimizing educational strategies in medical education.

## Materials and methods

This is a nested, single-center study that was hosted by the SET facility (AIIMS, Delhi). An informed consent form was obtained from all participants before the commencement of the study. Ethical approval was not required as no personal or identifiable information was collected or used.

### Course design

**FIGURE 1:**
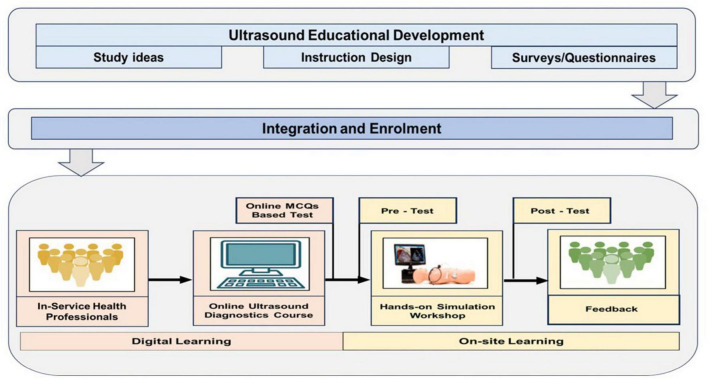
Illustration of iterative methodology adopted for designing, piloting, and scaling the ultrasound-guided diagnostic course and hands-on workshop. The model emphasizes needs-based curriculum development, pilot testing, evidence-informed refinement, and structured implementation with continuous feedback.

The course, structured using a blended learning approach, integrates online modules with practical, hands-on training sessions to strengthen learners’ understanding and competence in ultrasound diagnostics. It is led by 13 expert faculty members as illustrated in Figure 1. This course covers fundamental techniques and standardized protocols for the application of diagnostic ultrasound in emergency settings. The structure of the course is as follows.

#### Online component of the course

The online component of the training program encompasses core concepts, including machine handling, probe orientation, and image interpretation. Key modules covered foundation knowledge in cardiac, lung, abdominal, FAST scan, DVT, and ocular US. The e-learning package comprises 5 PDF resources and 6 instructional videos, each supplemented with 6–10 multiple-choice questions (MCQs) designed to reinforce learning. The completion of the MCQs requires approximately 4–5 min per session.

#### Hands-on component of the course

The training used high-fidelity ultrasound simulators that had modules for cardiac, lung, abdominal, vascular, and ocular ultrasound. The transthoracic echocardiography (TTE) simulator, equipped with a phased array transducer, enabled trainees to assess cardiac function, chamber size, valve integrity, and pericardial effusion through parasternal, apical, and subcostal views. Simulations of lung ultrasounds made it easier to find pneumothorax, pleural effusion, consolidation, and interstitial syndromes, with a focus on telling the difference between important artifacts like A-lines and B-lines. The abdominal module, using a curvilinear probe, supported training in general abdominal imaging and trauma scenarios, including FAST and eFAST protocols. Pathologies such as free fluid, hemothorax, and pericardial effusion were simulated for trauma assessment. Additionally, volunteer-based scanning allowed hands-on experience in DVT assessment via venous compressibility (femoral and popliteal veins) and ocular ultrasound for visualizing orbital structures.

### Participant allocation and equipment

The study was open to all in-service medical healthcare professionals involved in clinical care and training activities. The sample size was determined by the total number of eligible participants who enrolled during the study period, as the study aimed to evaluate program implementation rather than test a statistical hypothesis. Participants represented a broad spectrum of academic and non-academic cadres, including junior and senior residents. The departments invited for participation were categorized as follows: surgical and allied specialties like gastro-intestinal surgery, urology, gynecology and obstetrics, etc.; Anesthesiology; Critical Care and Perioperative Care; Emergency Medicine; Oncology and Supportive Care, General medicine and all allied specialities and super specialities like geriatric medicine, pulmonary medicine, nephrology, gastro-enterology, etc. For the hands-on practice sessions, participants were divided into two groups: the simulator-based group and the volunteer-based group. The participants were equally divided at each station. These sessions covered cardiac, lung, abdominal, and FAST scan USG techniques, with a 15-min time required by each group (followed by group rotation). DVT and ocular USG sessions are conducted on volunteers, each requiring a 20-min timeline (for each volunteer group). The training program was conducted using high-fidelity ultrasound simulators using CAE Vimedix (VIM-512) and CAE Vimedix Abdominal Ultrasound Simulator platform (CAE Healthcare, Canada).

### Online questionnaire/assessment tool

Online learning was followed by a structured multiple-choice question (MCQ)-based assessment administered at the end of each course topic to reinforce key concepts and evaluate topic-wise understanding. During the in-person workshop, assessments were administered using Google Forms to gauge baseline knowledge in the form of pre- and post-tests. The pre-test helped identify the participant’s initial understanding of core ultrasound concepts, while the post-test measured the knowledge gained after the blended learning and hands-on training components. All MCQs were developed and reviewed by a panel of subject experts and course faculty to ensure content validity, alignment with learning objectives, and appropriate level of difficulty. The ultrasound knowledge assessment tool was systematically structured to evaluate key domains essential for clinical ultrasound competency. It was divided into four main categories: Basic Principles and Physics, assessing foundational understanding of ultrasound wave behavior, Doppler principles, and image formation; Instrumentation and Probe Use, focusing on the appropriate selection and handling of transducers for various clinical contexts; Image Interpretation and Artifacts, evaluating the ability to identify normal and pathological findings, as well as recognize common ultrasound artifacts; and System-Specific Clinical Applications, which tested applied knowledge across organ systems, including hepatobiliary, renal, cardiovascular, pulmonary, pelvic, and vascular imaging.

### Data collection

The interventions needed in the course were collected in sequential phases aligned with a design-based research framework for formal data collection to iteratively develop, evaluate, and refine the blended learning program.

#### Online learning phase

Participants engaged with self-directed online modules hosted on the SARAL platform. Each module concluded with a formative assessment in the form of multiple-choice questions (MCQs) to evaluate knowledge acquisition. Module-wise scores were recorded to monitor learning progression. Additionally, structured online feedback was collected to assess content clarity, platform usability, and relevance to clinical practice. This feedback informed iterative refinement of instructional content and delivery.

#### Pre-workshop assessment phase

Before the hands-on simulation workshop, a structured pre-assessment was administered to evaluate baseline knowledge and procedural understanding. This step was essential for identifying learning gaps and tailoring the workshop to address specific needs.

#### Hands-on simulation workshop phase

Participants attended a simulation-based, instructor-led workshop focused on ultrasound-guided diagnostic and procedural skills. This immersive session allowed for real-time skill application and faculty-guided correction.

#### Post-workshop assessment and feedback phase

Immediately following the workshop, a post-assessment was conducted to evaluate the improvement in knowledge and procedural competence. In addition, participants completed a structured feedback form to capture their experience, confidence gain, and perceptions of training effectiveness.

### Statistical analysis

All statistical analyses and graphs were prepared using GraphPad Prism Software. Descriptive statistics were computed to summarize participant demographics. For online module performance, mean percentage scores and standard deviations were calculated for each module to assess comprehension levels and identify variability across topics. A subgroup analysis was conducted on participants who completed all seven modules (*n* = 18), comparing their performance to the broader cohort to examine the potential impact of curriculum completion on learning gains. While no formal inferential testing was applied for inter-module comparison due to sample size limitations, descriptive statistics were used to identify trends in learner performance and instructional effectiveness. For evaluating the effectiveness of the design implementation, pre-test and post-test scores were compared using an unpaired two-tailed Student’s *t*-test. A *p*-value of less than 0.05 was considered statistically significant, while values below 0.01 were regarded as highly significant.

## Results

**FIGURE 2:**
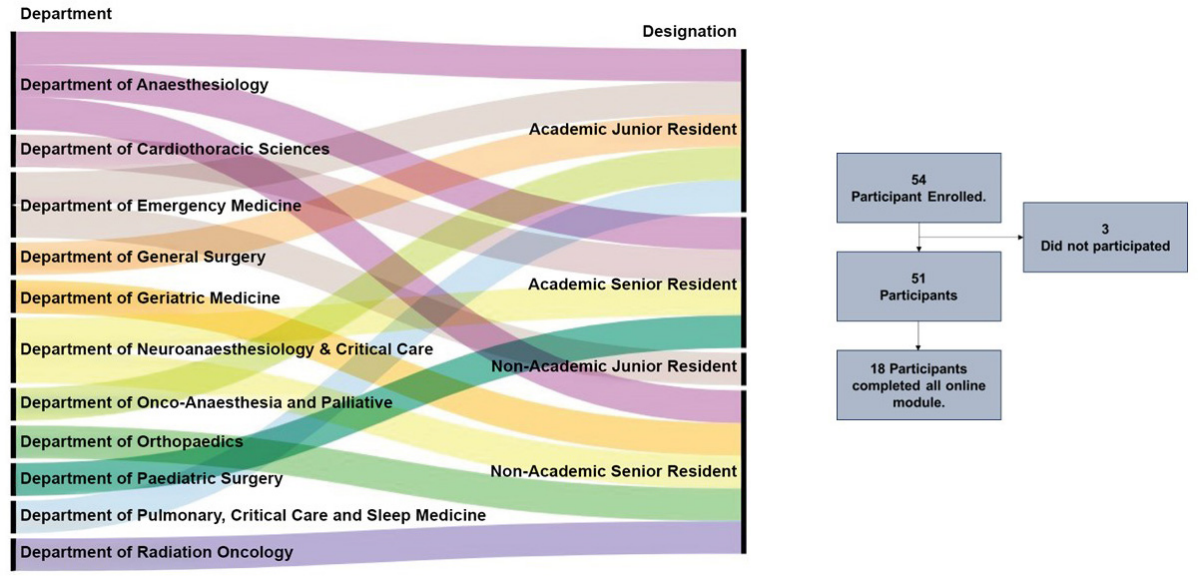
Demographic profile of course participants. Sankey plot depicting the demographic characteristics of the in-service healthcare participants, including their designation and departmental affiliations.

Fifty-four in-service medical healthcare professionals participated in the current study. The cohort included both academic and non-academic healthcare professionals involved in clinical care and teaching. 61.1% male and 38.9% female participants were from various clinical departments, as given in Figure 2. This highlights the academic alignment of residents across specialties, with the majority being from the department of anesthesiology. It is consistent with the routine use of ultrasound in perioperative and critical care settings, where point-of-care imaging is integral to clinical practice.

### Learning outcomes of the online course to guide design decisions

**FIGURE 3:**
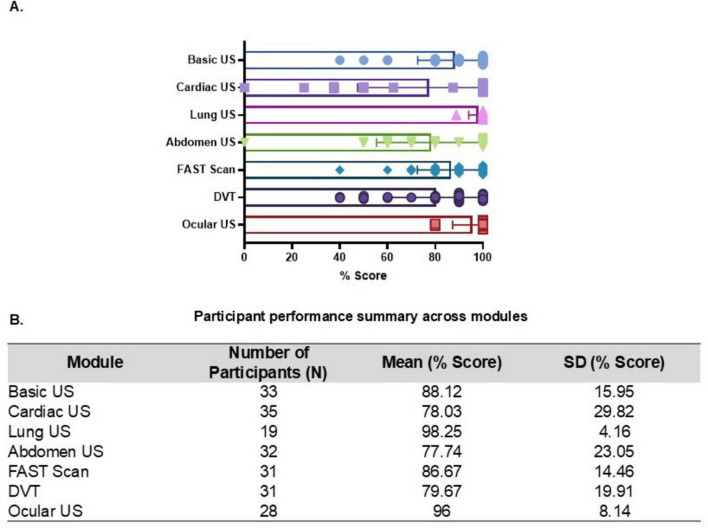
Performance in online ultrasound modules. **(A**) Bar graph showing the mean percentage scores achieved by participants in each online ultrasound module, highlighting areas of higher and lower performance. **(B)** Table summarizing the number of participants (*N*), mean scores, and standard deviations (SD) for each module, illustrating the distribution and consistency of participant performance across the courses on ultrasound-guided procedures.

The learner’s performances across seven online ultrasound modules were analyzed using mean percentage scores is as shown in Figures 3A,B. The lung and ocular US modules demonstrated the highest average scores (98.25 and 96.00%, respectively), with low standard deviations, indicating that these topics were well-understood and consistently performed. The cardiac US module, on the other hand, had the lowest average score (78.03%) and the most variation, which suggests that the participants were less familiar with or skilled at it. The Basic US and FAST Scan online modules also got rather high average results (88.12 and 86.67%, respectively), which shows that the students had a good understanding of the basics and were good at scanning for emergencies. The average scores for the abdominal US and DVT scanning modules were 77.74 and 79.67%, respectively, with moderate variability. This means that there are places where more practice may be helpful.

**FIGURE 4:**
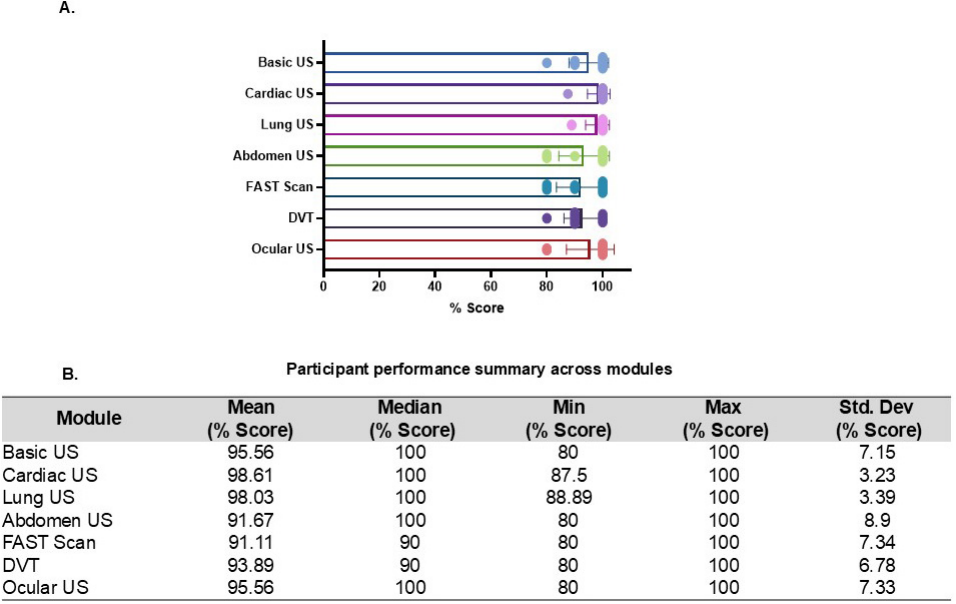
Performance of a subgroup (*n* = 18) completing all online ultrasound modules. **(A)** Bar plot showing the mean percentage scores for each ultrasound module among participants who completed the entire series, highlighting patterns of performance across modules. **(B)** Table presenting descriptive statistics for each module, including mean, median, minimum, maximum, and standard deviation (SD), representing the distribution and consistency of scores within this subgroup of participants.

The 18 participants who completed all modules consistently outperformed the broader cohort, with a mean score above 90% for each US training module as represented in Figure 4A. Significant improvements were seen in cardiac US and DVT scanning module for this subset of the participants. The cardiac US module demonstrated the highest mean score of 98.61% along with the lowest standard deviation (SD = 3.23%), indicating consistently high performance among participants. Ocular US (mean = 95.56%), DVT scanning (mean = 93.89%), and FAST scan (mean = 91.11%) modules also demonstrated high levels of performance with only variability (SDs ranging from 6.78 to 7.34%), reinforcing the overall success of the training. The basic and abdominal US online modules also showed an increase in learning percentage for this subset of the participants. However, abdominal US module had the lowest mean score (91.67%) and the highest standard deviation (SD = 8.90%) among all modules, suggesting that despite a high median (100%), a few students struggled more with this content, possibly due to anatomical complexity or variability in hands-on exposure suggesting that completing the curriculum supports the consolidation of foundational and systemic imaging skills (Figure 4B).

**FIGURE 5:**
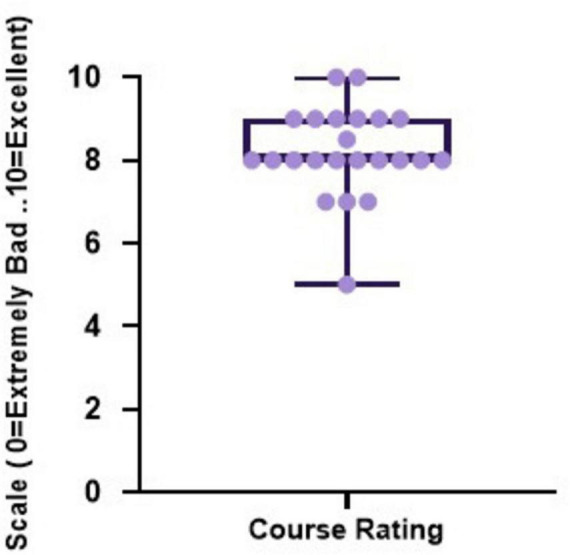
Overall feedback on the online ultrasound training program. Dot-box plot representation of participant’s overall satisfaction and variation in perceptions of the online course modules on a scale from 1 to 10, where 1 represents “extremely poor” and 10 represents “excellent.”

The overall online feedback is highlighted in Figure 5, which shows appreciation for the quality and clarity of presentations. The course was highly effective in meeting its learning objectives, with participants confirming its success. Participants described the course as “*very helpful,” “comprehensive,” and “well-designed,*” with multiple responses highlighting that it covered “*many left-over topics of POCUS*” and provided “*a basic understanding of key areas.*” Words like “*excellent,” “informative,*” and “*adequate*” appeared frequently, reflecting overall satisfaction with both content and structure. Moreover, when asked about the accessibility of the USG Course on SARAL, the response was overwhelmingly positive. This feedback revealed that the course’s success was in both content delivery and platform accessibility.

Several participants suggested various forms of enhancements when asked “*how to further enrich the learning experience?*” A common recommendation was the inclusion of more instructional videos, particularly to supplement or accompany PowerPoint slides where either was lacking. Some respondents also noted technical concerns, such as errors in quiz question options due to misaligned choices or incorrect answers, emphasizing the need for thorough proofreading. A few participants expressed a desire for access to downloadable presentations, additional handouts, and the inclusion of pathology-based visuals and more echocardiography-related content. Suggestions also included providing answer rationales for quiz items and updating video content to cover essential topics like eFAST. Overall, the course was regarded positively, with participants indicating it was a valuable and engaging learning resource.

### Pre-test and post-test assessment of knowledge improvement during the hands-on workshop and design decisions

**FIGURE 6:**
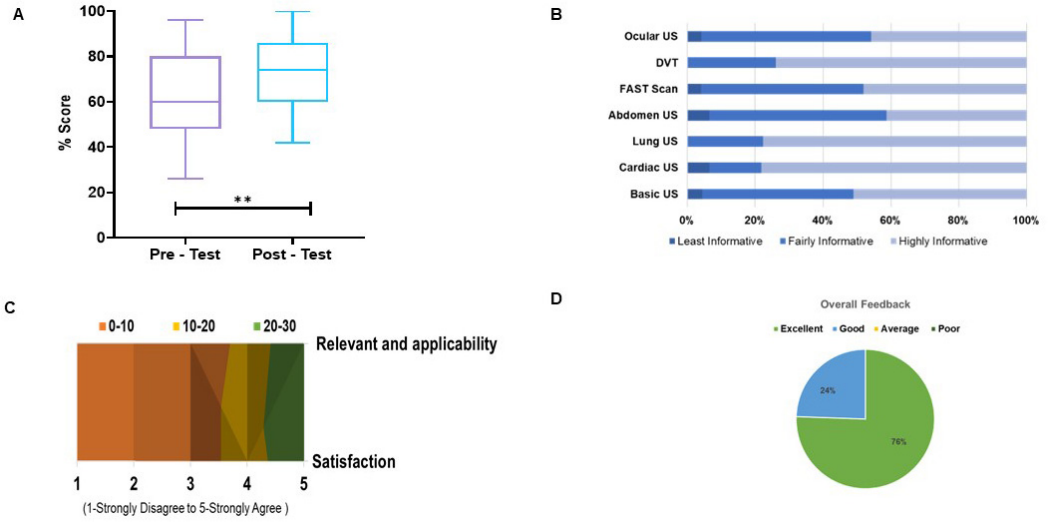
Pre/post-test performance assessment and perception of the hands-on simulation workshop quality (Number of participants = 51). **(A)** Box plot showing the distribution of percentage scores in the pre-test and post-test of the ultrasound training program showing improvements in participant performance. **(B)** Composite bar chart presenting participant feedback on each module, with modules on the X-axis illustrating differences in perceptions across the course components. **(C)** Surface chart illustrating participant responses on a 5-point Likert scale, capturing perceptions of relevance and satisfaction in the training program. **(D)** Pie chart depicting the overall feedback on the workshop providing a snapshot of participant satisfaction.

Pre-test and post-test score evaluations were compared by applying an unpaired *t*-test on participants (due to unmatched pre- and post-test responses data) who underwent the simulation-based training in ultrasound-guided techniques in Figure 6A. The results revealed a statistically significant improvement in post-test scores (Mean = 72.75%) compared to pre-test scores (Mean = 62.51%), with a mean difference of 10.24 ± 3.397 (*p* = 0.0033). The median score increased from the pre-test to the post-test, with a visibly higher interquartile range and upper whisker, indicating an overall enhancement in performance. The effect size, indicated by η^2^ (*R*^2^ = 0.08322), suggests a small to moderate practical significance. An *F*-test for equality of variances showed no significant difference between the two groups [*F*(50, 50) = 1.567, *p* = 0.1154], supporting the assumption of homogeneity of variance. Figure 6B depicts the participant’s perception of the informativeness of different US course modules.

As shown in the distribution plot (Figure 6C), most respondents selected positive options on the 5-point Likert scale (1-Strongly Disagree to 5-Strongly Agree), with the majority falling into the “Strongly Agree” and “Agree” categories (green). Neutral responses (yellow) were limited, and negative ratings (“Disagree” and “Strongly Disagree”—orange) were minimal. The visual distribution of participant ratings reveals a strong skew toward higher scores, with the majority of responses falling within the green zone, indicative of excellent or competent performance. This suggests that participants largely perceived the US-guided diagnostics training as highly effective, contextually relevant, and a reliable means of skill development. The overall feedback in Figure 6D reveals that 76% of participants provided positive responses, indicating strong satisfaction with the intervention. 24% gave neutral or moderate feedback, while no participants reported overall negative feedback. These results suggest a generally favorable reception of the US-guided diagnostics training program, with a substantial majority affirming its value and effectiveness.

Feedback from the participants after attending the hands-on ultrasound-guided procedures workshop included increasing hands-on training time, improving participant-to-machine ratios, and providing pre-course instructional videos and printed learning aids, as shown in Table 1. Content enhancements were suggested, such as modules on ejection fraction, airway ultrasound, and interventional procedures, with a clearer distinction between basic and advanced levels. Minor concerns were raised about technical access and alignment of assessments with course content. Overall, the workshop was well-received and considered highly valuable.

**TABLE 1:** Feedback-driven refinement guiding interventions to enhance the course design and delivery of the ultrasound-guided diagnostic training module.

Category	Feedback	Number of participants	Resulting intervention
Hands-on practice time	Requests for more hands-on time, longer duration per station, more interaction, dedicated time, and increased access to machines and volunteers	17	Expanded simulation scenarios; aligned hands-on cases with online modules; integrated interactive checkpoints
Pre-workshop preparation	Suggestion to provide instructional videos before the workshop	1	Pre-workshop instructional videos for self-paced learning
Learning materials and resources	Requests for booklets/printouts, handouts, take-home ready reckoner	3	Provided supplementary materials and ready reckoners; included references in online modules
Workshop frequency and continuation	Requests to conduct such workshops more frequently or regularly	4	Increased frequency of sessions; created a recurring schedule of workshops
Advertisement and awareness	Need for better communication and publicity within hospital premises	1	Improved internal communications and awareness campaigns
Content enhancement	Additions like ejection fraction assessment, airway ultrasound, advanced modules, interventional procedures, pathologies, grouping based on level (basic/advanced). Requests for a better explanation of basics, waveform teaching, and cardiac 2D echo for all	15	Introduced scaffolded learning, annotated video walkthroughs, and advanced content modules
Technical/logistical issues	Discrepancy in outline, system access problems	2	Revised module outlines; resolved system access issues
Overall positive feedback	“Excellent,” “great,” “good,” “awesome,” “very informative”	7	Reinforced strategies used in well-received modules as templates for other modules
No suggestions	Responded with “none” or “nil”	2	–

### Phase-wise outcomes of the design-based research implementation in the ultrasound-guided diagnostic training program

The design-based refinements are given in Table 2 by analyzing the program’s outcomes, variability in performance, and qualitative feedback. These targeted interventions aim to strengthen cognitive engagement, standardize content delivery, and enhance clinical applicability across modules. This iterative, data-informed refinement process reflects a design-based research approach to curriculum enhancement. By addressing cognitive complexity, learner variability, and simulation fidelity, the interventions aim to optimize the educational impact of each module. These steps ensure that both content delivery and hands-on training are tailored to support progressive skill acquisition, clinical reasoning, and learner engagement across the spectrum of ultrasound-guided diagnostics and interventions.

**TABLE 2:** Modifications to improve ultrasound training effectiveness.

Overall outcomes	Design-based intervention	Steps to overcome shortcomings
Consistently high scores with low variability in lung and ocular US modules	Hold on to instructional strategies (clarity, structure, visuals) used in these modules as models for other topics	Use these modules as templates to redesign complex modules (e.g., cardiac and abdominal US); document best practices
Low mean scores and high variability in cardiac US	Introduce scaffolded learning architecture; include clinical vignettes with annotated video walkthroughs; support iterative progression with embedded feedback	Redesign module using sequential content delivery, interactive checkpoints, and formative assessments with automated feedback
High variability in abdominal US despite good performance	Review module visuals and narration clarity; enhance anatomical illustrations and imaging-video correlation	Revise content with clearer image labeling, real patient case clips, and interactive cross-sections
Participants completing all modules performed better overall	Encourage full module completion via nudges, incentives, and progress reminders	Integrate milestone badges and reminders; require completion of modules before hands-on sessions
The pre-test showed moderate baseline knowledge	Enhance the diagnostic utility of pre-test by increasing cognitive demand	Develop a more rigorous pre-assessment with increased cognitive complexity by incorporating case-based and problem-solving items to better differentiate baseline competency levels
Simulation training led to statistically significant improvement	Retain simulation as a core training component; align hands-on cases with online modules	Expand simulation scenarios; ensure fidelity to online learning topics; explore video-recorded feedback for debrief
Variability in perception of module informativeness (e.g., cardiac, DVT)	Investigate the disconnect between online learning and simulation realism	Improve tactile simulation realism (e.g., with higher-fidelity phantoms); collect feedback via structured surveys
Ongoing feedback informed curriculum refinement	Continue feedback integration as a DBR cycle element	Create a live feedback dashboard for instructors; conduct quarterly curriculum reviews based on learner data

## Discussion

This design-based research approach demonstrates the effectiveness of a blended learning approach incorporating technology-enhanced simulation in ultrasound education for in-service medical professionals. The findings demonstrate that this pedagogical approach significantly improved on the foundational knowledge and confidence across various US domains. Elendu et al. demonstrated that immediate and structured feedback significantly enhances learning, thereby reinforcing the role of simulation as an invaluable tool in medical education (11). The ultrasound-guided diagnostic training module was iteratively refined following the principles of design-based research, whereby learner feedback directly informed curricular modifications. As detailed in Table 1, participants highlighted needs for increased hands-on practice, enhanced pre-workshop preparation, enriched learning materials, and advanced content coverage. These insights guided targeted interventions (Table 2), including scaffolded learning, interactive checkpoints, annotated video walkthroughs, and high-fidelity simulation aligned with online modules. Modules with low performance and high variability, such as cardiac and abdominal ultrasound, were redesigned to address specific gaps, while effective strategies from high-performing modules were adopted as templates. This feedback-driven approach demonstrates a clear causal link between qualitative input and iterative curriculum improvements.

Integrating asynchronous e-learning with high-fidelity simulation indeed raises important questions about balancing theoretical knowledge with hands-on practice and ensuring transfer of learning into clinical settings. To address this, we propose incorporating a structured assessment phase using Direct Observation of Procedural Skills (DOPS), which will evaluate whether residents can effectively translate online-acquired knowledge and simulation-based training into real-world. With the advances in the technologies including the use of image segmentation to create layered, anatomically accurate US datasets enabling learners to appreciate the cross-sectional anatomy better such as image segmentation hold promise in strengthening ultrasound education (12, 13). Segmentation algorithms that automatically delineate anatomical structures (e.g., liver, vessels, fetus) can provide learners with immediate visual feedback during training, thereby reducing cognitive load and facilitating pattern recognition (14–16). In parallel, the integration of artificial intelligence (AI) offers significant potential to further personalize learning by providing automated, real-time feedback and objective assessment of ultrasound performance metrics such as probe handling, image optimization, and anatomical identification. Such AI-enhanced analytics could complement instructor feedback, ensure standardization in evaluation, and support adaptive learning pathways that tailor the training experience to individual learner needs. However, it’s crucial to recognize that it’s not a one-size-fits-all solution for all training needs (17).

Our evaluation strategy, combining online module analytics with pre-test and post-test comparisons, enabled a comprehensive assessment of educational impact across a range of ultrasound applications. Interestingly, the pre-test scores across the cohort were relatively high, which could be due motivated learner population with prior exposure or self-initiated learning via online course module. This also signals the potential ceiling effect in assessing learning gains using conventional pre/post-test models (18). This observation necessitates the redesign of the curriculum with advanced modular content, scenario-based assessments, and emphasis on higher-order skills such as clinical integration and image-guided intervention planning.

The analysis revealed marked improvement in learner performance following the hands-on simulation workshop, especially in modules such as lung and ocular ultrasound. These findings support existing literature highlighting simulation’s ability to enhance learner confidence, provide immediate feedback, and reinforce correct technique through repetition and guided practice (19). In contrast, lower average scores and high variability in the cardiac ultrasound module point to possible instructional gaps or higher baseline complexity. This aligns with prior research indicating that cardiac ultrasound is often perceived as more technically demanding, requiring more structured scaffolding and supervised practice (20, 21). From a clinical standpoint, the relevance of ultrasound-guided interventions is particularly critical in procedures (22). Focused study on diagnostic US, its principles can be extrapolated to interventional training domains, where structured curricula are still evolving (23). Altogether, our study contributes to the growing body of evidence advocating for and implementing the integration of simulation and digital platforms into competency-based medical education.

Importantly, the subgroup of participants who completed all seven modules consistently outperformed the broader cohort, particularly in the more challenging cardiac and DVT modules. This finding supports the premise of competency over time-based progression, i.e., full engagement with the curriculum fosters deeper learning, improved retention, and enhanced skill acquisition (24). Similar to the findings of Boal et al., robotic surgery training using a modular curriculum with defined performance benchmarks (M-GEARS scores ≥ 80%) enabled a structured, stepwise progression. Trainees were allowed to advance only after demonstrating proficiency in foundational modules (25).

In alignment with the iterative, needs-responsive ethos of design-based research, participants emphasized cardiac and lung ultrasound as priority learning areas, reflecting their critical relevance in acute care (26). This study adopted a design-based approach, where curriculum elements were iteratively developed, piloted, and refined in response to both learner performance and contextual needs (27). The simulation environment functioned as a designed learning space, providing structured opportunities for repetition, feedback, and scaffolded performance elements aligned with cognitive apprenticeship and deliberate practice theories (28). Participants also highlighted DVT and trauma protocols as valuable skills, which corresponds with previous findings that simulation-based training improves procedural confidence and accuracy in vascular and emergency ultrasound. Improved understanding of probe orientation and machine knobology supports literature suggesting that technical mastery is foundational to effective ultrasound performance, fostering both skill acquisition and clinical readiness.

### Limitations

While the findings are promising, several limitations are present, such as that learner motivation was not assessed. It was assumed that participants were highly motivated, but no formal assessment of familiarity with ultrasound and learner motivation or self-efficacy was conducted, which may influence engagement and performance. As this study was conducted at a tertiary academic center equipped with high-end simulation facilities, the findings may not be directly generalizable to district hospitals with fewer resources. In addition, the outcomes of Design-Based Research (DBR) are intrinsically contingent upon the clinical milieu in which they are implemented. Variability in institutional practices, resource allocation, and the prevailing professional culture can substantially modulate both the feasibility and the educational impact of such interventions. Longitudinal outcomes such as skill retention, transfer to clinical practice, and patient impact were not evaluated. While the present study focused on short-term educational effectiveness, future longitudinal studies will be needed to establish the translation of these gains into clinical performance and patient safety. High pre-test scores in some modules may have limited measurable post-test gains, calling for more nuanced assessment tools and tiered content levels.

## Conclusion

This study reaffirms the utility of a design-based research approach incorporating blended learning for education in-service healthcare providers. Simulation-based blended learning offers a powerful tool for ultrasound education, combining safety, structure, and realism. While learner performance was strong across most modules, the data suggest the need for curriculum enhancements in some modules. It demonstrates significant short-term knowledge gains and provides evidence that module structure and learner engagement critically influence outcomes. Future research should focus on long-term competency retention, the impact of individualized learning trajectories, and broader implementation across specialties, particularly in interventional and image-guided disciplines.

## Data Availability

The raw data supporting the conclusions of this article will be made available by the authors, without undue reservation.

## References

[1] Stoumpos A Kitsios F Talias M. Digital transformation in healthcare: technology acceptance and its applications. *Int J Environ Res Public Health*. (2023) 20:3407. 10.3390/ijerph20043407 36834105 PMC9963556

[2] Vijayan S Kattuparambil J Mani P Chirukandath R Antony M. Juxtaposing pedagogical paradigms: the efficacy of Peer-assisted learning (PAL) versus Faculty-assisted learning (FAL) in the refinement of surgical proficiency. *BMC Med Educ*. (2025) 25:290. 10.1186/s12909-025-06827-2 39984895 PMC11846272

[3] Herrera-Aliaga E Estrada L. Trends and Innovations of simulation for twenty first century medical education. *Front Public Health*. (2022) 10:619769. 10.3389/fpubh.2022.619769 35309206 PMC8929194

[4] Bochatay N Ju M O’Brien B van Schaik SM. A scoping review of interprofessional simulation-based team training programs. *Simul Healthc*. (2024) 20:33–41. 10.1097/SIH.0000000000000792 38526045 PMC11776884

[5] Baker A Monuteaux M Mullan P Nagler J Dorney K. Simulation-based training in clinical event debriefing improves leadership performance. *Pediatr Emerg Care*. (2024) 41:86–93. 10.1097/PEC.0000000000003264 39509323

[6] Gibbs V. The role of ultrasound simulators in education: an investigation into sonography student experiences and clinical mentor perceptions. *Ultrasound*. (2023) 23:204–11. 10.1177/1742271X15604665 27433260 PMC4760595

[7] Wang Y. Design-based research on integrating learning technology tools into higher education classes to achieve active learning. *Comput Educ.* (2020) 156:103935. 10.1016/j.compedu.2020.103935

[8] Sekhavati P Wild T Martinez I Dion P Woo M Ramlogan R Instructional design features in ultrasound-guided regional anaesthesia simulation-based training: a systematic review. *Anaesthesia*. (2025) 80:572–81. 10.1111/anae.16527 39762010 PMC11987786

[9] Koivisto J Haavisto E Niemi H Haho P Nylund S Multisilta J. Design principles for simulation games for learning clinical reasoning: a design-based research approach. *Nurse Educ Today*. (2018) 60:114–20. 10.1016/j.nedt.2017.10.002 29096383

[10] Vallée A Blacher J Cariou A Sorbets E. Blended learning compared to traditional learning in medical education: systematic review and meta-analysis. *J Med Internet Res*. (2020) 22:e16504. 10.2196/16504 32773378 PMC7445617

[11] Elendu C Amaechi D Okatta A Amaechi E Elendu T Ezeh C The impact of simulation-based training in medical education: a review. *Medicine*. (2024) 103:e38813. 10.1097/MD.0000000000038813 38968472 PMC11224887

[12] Ansari M Mohanty S Mathew S Mishra S Singh S Abinahed J Towards developing a lightweight neural network for liver CT segmentation. In: Su R Zhang Y Liu H Frangi A editors. *Medical Imaging and Computer-Aided Diagnosis. MICAD 2022. Lecture Notes in Electrical Engineering.* (Vol. 810), Singapore: Springer (2022) 10.1007/978-981-16-6775-6_3

[13] Ansari M Mangalote I Masri D Dakua S. Neural network-based fast liver ultrasound image segmentation. *Proceedings of the International Joint Conference on Neural Networks.* Piscataway, NJ: IEEE (2023). 10.1109/JBHI.2024.3502694

[14] Dakua S. Performance divergence with data discrepancy: a review. *Artif Intell Rev.* (2013) 40:429–55. 10.1007/s10462-011-9289-8

[15] Dakua S Sahambi J. Automatic left ventricular contour extraction from cardiac magnetic resonance images using cantilever beam and random walk approach. *Cardiovasc. Eng*. (2010) 10:30–43. 10.1007/s10558-009-9091-2 20082140

[16] Dakua S Sahambi J. Detection of left ventricular myocardial contours from ischemic cardiac MR images. *IETE J Res.* (2011) 57:372–84. 10.4103/0377-2063.86338

[17] Dale-Tam J Thompson K. Evolution of a simulation design template at a canadian academic hospital. *Clin Simul Nurs.* (2022) 73:17–20. 10.1016/j.ecns.2022.10.001

[18] Savage A McNamara P Moncrieff T O’Reilly G. Review article: e-learning in emergency medicine: a systematic review. *Emerg Med Australas*. (2022) 34:322–32. 10.1111/1742-6723.13936 35224870 PMC9306619

[19] Rong K Lee G Herbst M. Effectiveness of near-peer versus faculty point-of-care ultrasound instruction to third-year medical students. *POCUS J*. (2022) 7:239–44. 10.24908/pocus.v7i2.15746 36896384 PMC9983727

[20] Beck S Whalley G Coffey S Hawley A Anakin M. Cardiac ultrasound training for medical students utilizing drawing and ward-based instruction. *MedEdPORTAL*. (2025) 21:11485. 10.15766/mep_2374-8265.11485 39802645 PMC11717889

[21] Brown K Riley A Alade K Kyle W Castro D Tcharmtchi M A novel tool for teaching cardiac point-of-care ultrasound: an exploratory application of the design-based research approach. *Pediatr Crit Care Med*. (2020) 21:e1113–8. 10.1097/PCC.0000000000002441 32701750

[22] Choi W Cho Y Ha Y Oh J Lee H Kang B Role of point-of-care ultrasound in critical care and emergency medicine: update and future perspective. *Clin Exp Emerg Med*. (2023) 10:363–81. 10.15441/ceem.23.101 38225778 PMC10790072

[23] Teichgräber U Ingwersen M Ehlers C Mentzel H Redies C Stallmach A Integration of ultrasonography training into undergraduate medical education: catch up with professional needs. *Insights Imaging*. (2022) 13:1–8. 10.1186/s13244-022-01296-3 36153444 PMC9509508

[24] Jiang Z Wang J Chen X Li Y Ni D Zhu J Competency-based ultrasound curriculum for standardized training resident: a pre- and post-training evaluation. *BMC Med Educ*. (2024) 24:1516. 10.1186/s12909-024-06560-2 39709401 PMC11663331

[25] Boal M Afzal A Gorard J Shah A Tesfai F Ghamrawi W Development and evaluation of a societal core robotic surgery accreditation curriculum for the UK. *J Robot Surg*. (2024) 18:305. 10.1007/s11701-024-02062-x 39106003 PMC11303427

[26] Moore C Copel J. Point-of-care ultrasonography. *N Engl J Med*. (2011) 364:749–57. 10.1056/NEJMra0909487 21345104

[27] Harih G Cretnik A. Interdisciplinary approach to tool-handle design based on medical imaging. *Biomed Res Int*. (2013) 2013:159159. 10.1155/2013/159159 24171159 PMC3792520

[28] Cassara M Schertzer K Falk M Wong A Hock S Bentley S Applying educational theory and best practices to solve common challenges of simulation-based procedural training in emergency medicine. *AEM Educ Train*. (2020) 4:S22–39. 10.1002/aet2.10418 32072105 PMC7011411

